# Lessons learned from terror attacks: thematic priorities and development since 2001—results from a systematic review

**DOI:** 10.1007/s00068-021-01858-y

**Published:** 2022-01-13

**Authors:** Nora Schorscher, Maximilian Kippnich, Patrick Meybohm, Thomas Wurmb

**Affiliations:** 1 Department of Anaesthesiology, Intensive Care, Emergency and Pain Medicine, University Hospital Wuerzburg, Wuerzburg, 97080, Germany; 2 Subsection Emergency and Disaster Relief Medicine, Department of Anaesthesiology, Intensive Care, Emergency and Pain Medicine, University Hospital Wuerzburg, Oberduerrbacherstrasse 6, 97080 Wuerzburg, Germany

**Keywords:** Terror attacks, Evaluation, Lessons learned, Emergency preparedness, Public health preparedness, Mass casualties

## Abstract

**Purpose:**

The threat of national and international terrorism remains high. Preparation is the key requirement for the resilience of hospitals and out-of-hospital rescue forces. The scientific evidence for defining medical and tactical strategies often feeds on the analysis of real incidents and the lessons learned derived from them. This systematic review of the literature aims to identify and systematically report lessons learned from terrorist attacks since 2001.

**Methods:**

PubMed was used as a database using predefined search strategies and eligibility criteria. All countries that are part of the Organization for Economic Cooperation and Development (OECD) were included. The time frame was set between 2001 and 2018.

**Results:**

Finally 68 articles were included in the review. From these, 616 lessons learned were extracted and summarized into 15 categories. The data shows that despite the difference in attacks, countries, and casualties involved, many of the lessons learned are similar. We also found that the pattern of lessons learned is repeated continuously over the time period studied.

**Conclusions:**

The lessons from terrorist attacks since 2001 follow a certain pattern and remained constant over time. Therefore, it seems to be more accurate to talk about lessons identified rather than lessons learned. To save as many victims as possible, protect rescue forces from harm, and to prepare hospitals at the best possible level it is important to implement the lessons identified in training and preparation.

## Introduction

### Background

The emergency management of terrorist attacks has been one of the prominent topics in disaster and emergency medicine before the SARS-CoV-2 pandemic. The most recent attacks have shown that this particular threat is still present and highly relevant today [1–4]. The idea of “stopping the dying as well as the killing”, which has been coined by Park et al. after the London Bridge and Borough Market attacks in 2017, emphasizes the urgent need to focus on emergency management and early medical and surgical intervention [5].

Rescue systems and hospitals must prepare themselves to manage terrorist attacks in order to save as many lives as possible and to return rescue forces from the missions unscathed. As it is impossible to conduct prospective, high-quality scientific studies, the definition of these medical and tactical strategies relies on the analysis of real incidents and the lessons learned derived from them. After the Paris terror attacks in 2015 for example, important publications, describing the events of the night of the 13th of November 2015, were published [6, 7]. Two publications, one by the French Health Ministry and one by Carli et al., about the “Parisian night of terror” have gone a step further and have clearly described the lessons learned from these attacks [8, 9]. Importantly, experts agree on the importance of the scientific and systematic evaluation of the most recent terror attacks [10]. Challen et al. proved the existence of a large body of literature on the topic in 2012 already, but questioned its validity and generalisability. The authors based their conclusion on a review, which focused on emergency planning for any kind of disaster [11].

More than ever, the principle applies, that the preparation for extraordinary disastrous incidents is the decisive prerequisite for successful management. The lack of preparedness for the SARS-CoV-2 pandemic has taught modern society this lesson.

With the aim to identify and systematically report the lessons learned from terrorist attacks as an important basis for preparation, we conducted the presented systematic review of the literature.

## Materials and methods

### Study design and search strategy

This is a systematic review of the literature with the focus on lessons learned from terror attacks. A comprehensive literature search was performed to identify articles reporting medical and surgical management of terrorist attacks and lessons learned derived from them. PubMed was used as database. The first search term concentrated on terrorism, the second on medical/surgical management and the third on evaluation and lessons learned. Adapted PRISMA guidelines were used and all articles were checked and reported against its checklist [12].

The search terms were formulated as an advanced search in PubMed in the following way: Search: ((Terror* OR Terror* Attack* OR Terrorism* OR Mass Casult* Incident* OR Mass Shooting* OR Suicide Attack* OR Suicide Bomb* OR Rampage* OR Amok*) AND (Prehospital* Care* OR Emergenc* Medical* Service* OR Emergenc* Service* OR Emergenc* Care* OR Rescue Mission* OR Triage* OR Disaster* Management* OR First* Respon*)) AND (Lesson* Learn* OR Quality Indicator* OR Evaluation* OR Analysis* OR Review* OR Report* OR Deficit* OR Problem*).

### Eligibility criteria and study selection

Time frame: The attack on the World Trade Centre in New York, the Pentagon in Arlington, and the crash of a hijacked airliner in 2001 is considered the event that brought international terrorism onto the world stage with the beginning of the new millennium. The attacks have been documented and analysed in great detail. For this reason, this analysis starts in 2001 and ends with the terrorist attacks in London and Manchester in 2017. The search history was extended to the year 2018.

Included countries: Terrorism is a worldwide phenomenon. Attempting to evaluate the data of all terrorist attacks that have occurred since 2001 seems impossible due to the extremely high number. The work therefore focuses mainly on Western-oriented democracies, for which a terrorist attack is still a relatively rare event and whose infrastructure and emergency services recently had to adapt to this challenge. The Organization for Economic Cooperation and Development (OECD)—countries therefore represent a reasonable selection of countries for this study.

Exclusion criteria:

1. Articles reporting mass casualty incidents without a terroristic background
2. Personal reports without any clear defined lessons learned
3. Articles dealing exclusively with chemical, biological, radiological and nuclear (CBRN) terrorism
4. Articles dealing with a narrow point of view and only dealing with specific types of injuries such as burns or psychiatry
5. Articles not written in English.

Articles dealing exclusively with chemical, biological, radiological and nuclear terrorism (CBRN-attacks) were excluded from the literature-search. The reason for this is the large number of special problems and issues associated with this type of incident. To address this adequately, a separate literature search would be necessary.

### Data abstraction

The lessons learned from each included article were extracted according to the inclusion and exclusion criteria. Duplicated data was excluded. As expected, there was a vast number of individual lessons learned. To summarize the results, it was imperative to divide them into categories. As a basis for developing the categories existing systems were used. The reporting system of Fattah et al. defines 6 categories, but these were not sufficient to represent all types of lessons learned [13]. Wurmb et al. had recently developed 13 clusters of quality indicators [14], some of which we were able to adopt. However, both systems focused on categories that serve to describe the overall setting of a rescue mission and were therefore not fully suitable for clustering lessons learned. Finally these 15 categories were used for clustering the lessons learned:

- Preparedness/planning/training
- Tactics/organisation/logistics
- Medical treatment and Injuries
- Equipment and supplies
- Staffing
- Command
- Communication
- Zoning and safety scene
- Triage
- Patient flow and distribution
- Team spirit
- Role Understanding
- Cooperation and multidisciplinary approach
- Psychiatric support
- Record keeping

After defining the categories, the lessons learned were assigned to them. Where applicable, the lessons learned were divided into “pre-incident”, “during incident” and “post-incident” within the different categories.

## Results

**Fig. 1:**
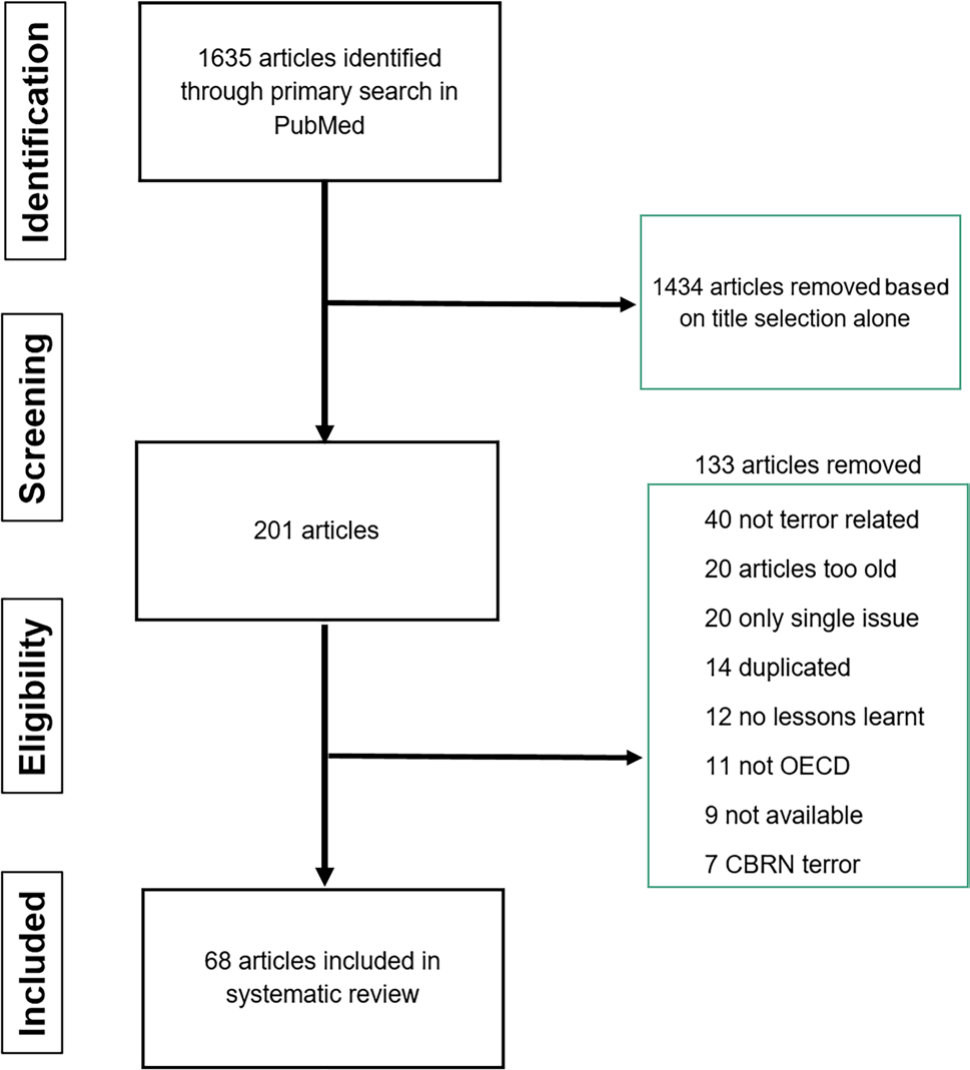
Process to identify the articles included in the systematic review

The extended PubMed Search yielded 1635 articles out of which 1434 articles were excluded on title selection only. The abstracts of the remaining 201 articles were evaluated and finally 68 articles were included in the analysis (Fig. 1).

To evaluate the quality of the included studies, the PRISMA evaluation was used and all articles were checked and reported against its checklist and then rated as either high quality (HQ), acceptable quality (AQ) or low quality (LQ) paper (Table 1) [12].

**Table 1:** Overview of all included articles with PRISMA evaluation

Authors	Year	Incident site	Study type	PRISMA
Roccaforte et al. [15]	2001	USA 9/11	Retrospective	AQ
Martinez et al.[16]	2001	USA 9/11	Eye Witness	AQ
Cook et al. [17]	2001	USA 9/11	Eye Witness	AQ
Tamber et al. [18]	2001	USA 9/11	Expert Opinion	AQ
Simon et al. [19]	2001	USA 9/11	Review/Report	AQ
Mattox et al. [20]	2001	USA 9/11	Review/Report	AQ
Shapira et al. [21]	2002	Israel	General Review	HQ
Frykberg et al. [22]	2002	Multiple	Review/Report	HQ
Garcia-Castrillo et al. [23]	2003	Madrid, Spain	Review/Report	AQ
Shamir et al. [24]	2004	Israel	Review/Report	HQ
Einav et al. [25]	2004	Israel	Guidelines	HQ
Almogy et al. [26]	2004	Israel	Review/Report	AQ
Rodoplu et al. [27]	2004	Istanbul, Turkey	Retrospective Study	AQ
Kluger et al. [28]	2004	Israel	Review/Report	AQ
Gutierrez de Ceballos et al. [29]	2005	Madrid, Spain	Retrospective Study	AQ
Kirschbaum et al. [30]	2005	USA 9/11	Lessons Learned	HQ
Aschkenazy-Steuer et al. [31]	2005	Israel	Retrospective Study	HQ
Lockey et al. [32]	2005	London, UK	Retrospective Study	HQ
Hughes et al. [33]	2006	London, UK	Review/Report	AQ
Shapira et al. [34]	2006	Israel	Review/Report	AQ
Aylwin et al. [35]	2006	London, UK	Review/Report	HQ
Mohammed et al. [36]	2006	London, UK	Review/Report	AQ
Bland et al. [37]	2006	London, UK	Personal Review	AQ
Leiba et al. [38]	2006	Israel	Review/Report	HQ
Singer et al. [39]	2007	Israel	Review/Report	HQ
Schwartz et al. [40]	2007	Israel	Review/Report	AQ
Gomez et al. [41]	2007	Madrid, Spain	Review/Report	AQ
Bloch et al. [42]	2007	Israel	Review/Report	AQ
Bloch et al. [43]	2007	Israel	Review/Report	AQ
Barnes et al. [44]	2007	London, UK	Government Evaluation	HQ
Carresi et al. [45]	2008	Madrid, Spain	Review/Report	HQ
Raiter et al. [46]	2008	Israel	Review/Report	HQ
Shirley et al. [47]	2008	London, UK	Review/Report	HQ
Almgody et al. [48]	2008	Multiple	Review/Report	AQ
Turegano-Fuentes et al. [49]	2008	Madrid, Spain	Review/Report	AQ
Pinkert et al. [50]	2008	Israel	Review/Report	HQ
Pryor et al. [51]	2009	USA 9/11	Review/Report	HQ
Lockey et al. [52]	2012	Utoya, Norway	Review/Report	AQ
Sollid et al. [53]	2012	Utoya, Norway	Review/Report	AQ
Gaarder et al. [54]	2012	Utoya, Norway	Review/Report	AQ
No authors listed [55]	2013	Boston USA	Review/Report	AQ
Jacobs et al. [56]	2013	USA	General Review	AQ
Gates et al. [57]	2014	Boston, USA	Review/Report	AQ
Wang et al. [58]	2014	Multiple	General Review	HQ
Ashkenazi et al. [59]	2014	Israel	Overall Review	AQ
Thompson et al. [60]	2014	Multiple	Retrospective	AQ
Rimstad et al. [61]	2015	Oslo, Norway	Retrospective	AQ
Goralnick et al. [62]	2015	Boston, USA	Retrospective	AQ
Hirsch et al. [6]	2015	Paris, France	Personal Review	HQ
Lee et al. [63]	2016	San Bernadino, USA	Personal Review	HQ
Pedersen et al. [64]	2016	Utoya, Norway	Review/Report	AQ
Raid et al. [65]	2016	Paris, France	Personal Review	AQ
Philippe et al. [8]	2016	Paris, France	Government Review	HQ
Traumabase et al. [66]	2016	Paris, France	Personal Review	HQ
Gregory et al. [67]	2016	Paris, France	Review/Report	AQ
Ghanchi et al. [68]	2016	Paris, France	Review/Report	AQ
Khorram-Manesh et al. [69]	2016	Multiple	Review/Report	HQ
Goralnick et al. [10]	2017	Paris/Boston	Expert Opinion	AQ
Lesaffre et al. [70]	2017	Paris, France	Review/Report	AQ
Wurmb et al. [71]	2018	Würzburg, Germany	Lessons Learned	HQ
Brandrud et al. [72]	2017	Utoya, Norway	Review/Report	HQ
Carli et al. [9]	2017	Paris/Nice, France	Review/Report	HQ
Borel et al. [73]	2017	Paris, France	Review/Report	AQ
Bobko et al. [74]	2018	San Bernadino, USA	Review/Report	AQ
Chauhan et al. [75]	2018	Multiple	Review/Report	HQ
Hunt et al. [76]	2018	London/Manchester, UK	Review/Report	HQ
Hunt et al. [77]	2018	London/Manchester, UK	Review/Report	HQ
Hunt et al. [78]	2018	London/Manchester, UK	Review/Report	HQ

*HQ* high quality, *AQ* acceptable quality, *LQ* low quality, *USA* United States of America, *UK* United Kingdom

**Fig. 2:**
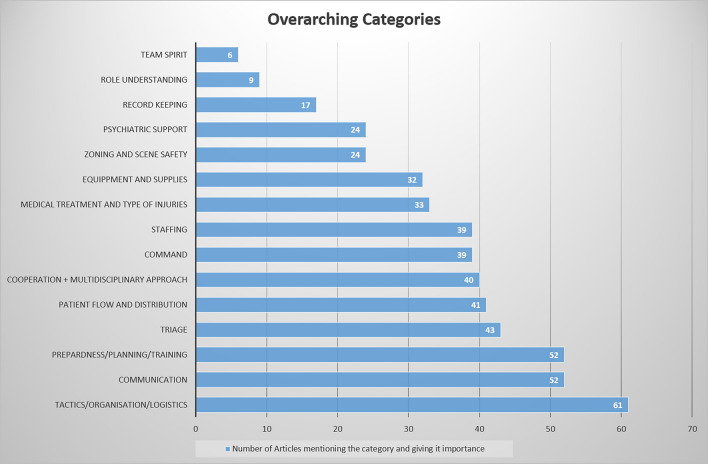
Number of articles mentioning each of the 15 categories

**Fig. 3:**
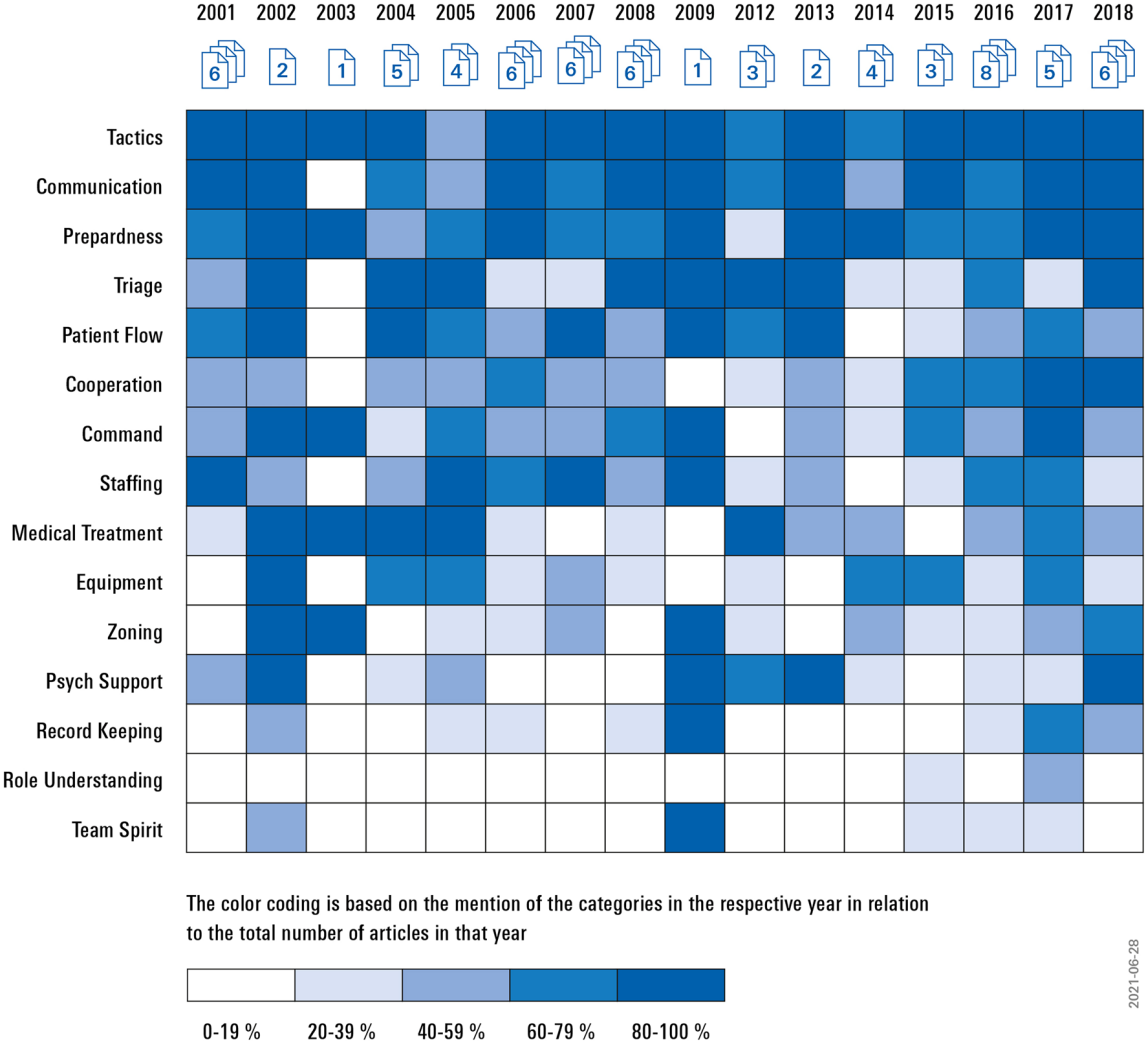
Categories of lessons learned from terror attacks—development since 2001

A total of 616 lessons learned were assigned to the 15 categories. If a lesson matched more than one category, it was assigned to all matching categories. Therefore, multiple entries occur in some cases. Table 2 shows the distribution of categories across all included articles, while Fig. 2 shows the number of articles in which each category appears. In this figure, the publications are assigned to the respective categories. This provides an overview of the number of articles dealing with each category. An overview of the distribution over time is later given in Fig. 3. Lessons learned within the category “tactics/organisation/logistics” were mentioned most frequently, while the category “team spirit” was ranked last in this list.

**Table 2:** Distribution of the 15 clusters across all included articles

Study	Year	1	2	3	4	5	6	7	8	9	10	11	12	13	14	15
Roccaforte et al. [15]	2001	x	x	x					x	x	x					
Martinez et al.[16]	2001	x	x		x	x	x	x	x			x				
Cook et al. [17]	2001		x		x	x		x	x	x		x				
Tamber et al.[18]	2001	x	x	x			x	x					x			
Simon et al.[19]	2001	x	x	x	x	x			x			x				
Mattox et al. [20]	2001	x	x	x		x	x		x							
Shapira et al. [21]	2002	x	x	x	x	x	x	x	x	x	x	x	x		x	
Frykberg et al. [22]	2002	x	x	x	x	x		x		x	x	x	x	x		
Garcia-Castrillo et al. [23]	2003	x		x				x		x			x			
Shamir et al.[24]	2004	x	x		x	x	x	x	x	x	x				x	
Einav et al. [25]	2004	x	x	x	x	x				x	x					
Almogy et al. [26]	2004	x		x					x	x	x					
Rodoplu et al. [27]	2004	x	x		x	x				x		x				
Kluger et al. [28]	2004	x			x	x	x	x								
Gutierrez de Ceballos et al. [29]	2005			x	x				x	x			x			
Kirschbaum et al. [30]	2005	x	x	x	x	x	x	x	x	x	x	x		x		
Aschkenazy-Steuer et al. [31]	2005	x		x	x	x	x	x	x	x	x					
Lockey et al. [32]	2005		x		x	x		x	x	x	x	x				
Hughes et al. [33]	2006	x	x	x			x				x			x		
Shapira et al. [34]	2006	x	x	x			x						x			
Aylwin et al. [35]	2006	x		x	x	x	x	x	x	x						
Mohammed et al. [36]	2006	x	x	x			x	x	x			x		x		
Bland et al. [37]	2006	x	x	x		x			x				x			x
Leiba et al. [38]	2006	x		x	x	x		x	x	x	x					
Singer et al. [39]	2007	x	x	x	x	x	x	x	x	x	x	x	x		x	
Schwartz et al. [40]	2007	x		x		x			x	x						
Gomez et al. [41]	2007	x	x				x	x	x	x	x					
Bloch et al. [42]	2007					x			x				x			
Bloch et al. [43]	2007	x	x	x	x	x			x							
Barnes et al.[44]	2007	x	x	x		x	x	x			x		x	x		
Carresi et al.[45]	2008	x	x	x	x		x	x	x	x				x		
Raiter et al.[46]	2008	x	x		x	x					x					
Shirley et al.[47]	2008	x		x			x	x		x			x		x	
Almgody et al. [48]	2008	x	x	x	x			x	x		x					
Turegano-Fuentes et al. [49]	2008	x	x		x	x	x		x					x		
Pinkert et al. [50]	2008	x	x	x	x	x		x								
Lockey et al. [52]	2012	x	x		x				x		x	x				
Sollid et al. [53]	2012	x	x		x	x					x	x				
Gaarder et al. [54]	2012			x	x	x	x			x	x		x			
NN et al. [55]	2013	x	x	x	x	x			x			x				
Jacobs et al. [56]	2013	x	x	x	x	x	x	x		x		x				
Gates et al. [57]	2014	x	x	x				x			x		x			
Wang et al. [58]	2014	x		x	x					x	x					
Ashkenazi et al. [59]	2014			x								x	x			
Thompson et al. [60]	2014	x	x	x			x			x	x					
Rimstad et al. [61]	2015	x	x	x				x								
Goralnick et al. [62]	2015	x	x				x	x			x				x	x
Hirsch et al. [6]	2015	x	x	x	x	x	x		x		x		x			
Lee et al. [63]	2016	x	x		x	x	x	x	x		x	x			x	
Pedersen et al. [64]	2016	x			x	x	x			x		x				
Raid et al. [65]	2016	x	x	x	x		x	x	x		x		x			
Philippe et al. [8]	2016	x	x	x	x		x	x		x				x		
Traumabase et al. [66]	2016			x					x				x		x	
Gregory et al. [67]	2016	x				x	x		x	x						
Ghanchi et al. [68]	2016	x	x	x	x			x	x							x
Khorram-Manesh et al. [69]	2016	x	x	x	x	x	x	x		x	x		x	x		
Goralnick et al. [10]	2017	x	x	x			x	x	x	x						x
Lesaffre et al. [70]	2017	x	x	x			x	x	x		x					
Brandrud et al. [72]	2017	x	x	x		x	x	x	x				x	x	x	x
Carli et al. [9]	2017	x	x	x	x	x	x	x		x	x	x	x	x		
Borel et al. [73]	2017	x	x	x	x	x	x	x	x	x	x			x		
Wurmb et al. [71]	2018	x	x	x	x	x	x					x		x		
Bobko et al. [74]	2018	x	x	x	x		x	x	x			x	x			
Chauhan al. [75]	2018	x	x	x	x	x	x			x	x	x				
Hunt et al. [76]	2018	x	x	x	x	x	x	x		x	x	x	x	x		x
Hunt et al. [77]	2018	x	x	x	x	x		x	x	x		x	x	x		
Hunt et al. [78]	2018	x	x	x			x						x	x		

1—Tactics/organization/logistics, 2—Communication, 3—Preparedness/planning/training 4—Triage, 5—Patient flow and distribution, 6—Cooperation/multi-disciplinary approach, 7—Command, 8—Staffing, 9—Medical treatment and type of injuries, 10—Equipment/supplies, 11—Zoning/scene safety, 12—Psych support, 13—Record keeping, 14—Role understanding, 15—Team spirit

To obtain a graphical overview over the entire study period, the frequency with which the categories were mentioned per year were colour-coded and presented in a matrix (Fig. 3).

A summary of all lessons learned assigned to the 15 categories can be found in Table 3.

**Table 3:** lessons learned assigned to the 15 overwhelming categories

Lessons learned	Tactics/organization/logistics
Pre-incident	
1	Offer a detailed manual for potential terror attacks
2	Need for having a solid disaster plan for each hospital
3	Have a national standard for major incidents and a preparedness concept/disaster response plan
4	Adequate trauma centre concepts on national level
5	Use trauma guidelines
6	Conduct updated disaster plans/drills
7	Active pre-planned protocols—pre hospital protocol + hospital protocol
8	All hospitals should be included in contingency planning
9	Do not base disaster plan on average surge rates
10	Standardisation in hospital incident planning
11	Have an emergency plan for preparedness
12	Use standard Protocols but keep flexibility
13	Establishment of various anti-terror contingency plans (hijack/bombing/shooting)
14	Mini disasters as basis for escalation (flu season)
15	Crisis management based on knowledge and data collection
During the incident	
16	Activate contingency/emergency plans soon
17	Organisation of trauma teams that stay with a patient
18	Cancellation of all elective surgery/discharge of all non-urgent patients
19	Establish a public information centre close to hospital
20	Alert all hospitals
21	Prehospital and hospital coordination + communication is necessary
22	Crowd control is important
23	Maximise surge capacity
24	Distance to hospital site is major distribution factor
25	Evacuation of the less critically ill to further away hospitals
26	Importance of controlled access to hospitals
27	Avoid main gate syndrome—overwhelmed resources at the closest hospital
28	Avoid overcrowding in the ER
29	Activation of white plan—all hospitals/all staff/empty beds → no shortage
30	Recruit help from outside early on
31	Do not forget flexibility
32	Combination of civil defence and emergency medical services
33	Designated treatment area
34	Rapid scene clearance—highly organised und efficient
35	Flexibility across incident sites/hospitals
36	Vehicle coordination and rapid accumulation
37	Set principles rather than fixed protocols to allow for flexibility
38	Importance of quick evacuation
39	Ambulance stacking area to allow access and reduce traffic jam
40	Important to declare major incident as soon as possible
41	Manage uncertainties and scene
42	Coordination of rescue—especially HEMS
43	Rapid logistical response
44	Divide emergency response into stages break into smaller parts
45	Adaptation of decisions taken
46	Early decision by incidence commander needed
47	No headquarter at frontline
48	Peri-incident intensive care management—forward deployment
49	Critical mortality is reduced by rapid advanced major incident management
50	Use ICU staff for resuscitation and triage
51	Four step approach to terror attacks: analysis of scenario; description of capabilities, analysis of gaps, development of operational framework
52	Experienced personnel should treat patient and not take on organisation
53	Empty hospital immediately
54	Focus on increasing bed capacity especially ICU beds
55	Constant update on resources and surge limitation of all hospitals
56	Trauma leaders must be aware of bed capacities
57	Combined activation of major incident plans (all EMS services)
58	Early activation of surge capacity
59	Crucial interaction/communication between hospital/police/municipalities
60	Fullback structures but flexibility and improvisation important
61	Tactical management—get an overview and do not get stuck in details
62	Prehospital damage control—military concepts in civilian setting
63	Regional resource mobilisation vital
64	Have a plan but use continuous reassessment and modification of response strategy
65	Use METHANE to assess incident
66	Clear escalation plan
67	Coordination and collaboration should be planned and practised at intra/inter-regional, multiagency and multiprofessional levels
68	Improved forensic management
69	Logistic is important for operational strategic roles
70	Maintaining access to other emergencies MI/stroke, etc.
71	Gradual De-escalation – part of contingency plan
72	Issue: recognition of situational aspect and severity + complexity—evolving risk
73	Cockpit view due to HEMS—helpful in big sweep of casualties
74	Limited mobilisation at remote hospitals
75	Incident commander appoints: liaison officer; public information officer; personnel officer; logistics officer; data officer; medical command officer; patient/family information officer
76	“ABCD response”: assess incident size and severity, alert backup personnel, perform initial casualty care, and provide definitive treatment
77	Authority and command structure—two command posts—administrational vs medical management
78	Med Students used as runners
79	Tape fixed with name/specialty
80	Delays should be expected
81	Disruption in transport—lengthens rescue effort
82	Guidelines on biochemical warfare
83	Structural organisation important
84	Clear and well-structured coordination
85	Management of uninjured survivors and relatives—good communication
86	Development of operational framework
87	Assessment and re-evaluation of disaster plans
88	ED as epicentre
89	Most senior emergency physician directs traffic/surgeons overseas area—triage not by most senior personnel
90	Volunteer surges difficult to manage but can be helpful
91	Need to increase morgue facilities
92	Improved alert system
93	Clear communication, organization and decision making skills
94	Robust and simple organisation and command
Post-incident	
95	Clinical representation at strategic level to facilitate cooperation between networks/regions
96	Support from neighbouring regions during terror
97	Develop a network of capacities and capabilities which is constantly updated
98	Gaps in provision of rehab services—acute phase vs long term phase
99	Access to legal and financial support for victims
100	Importance of evaluation and improvement of emergency plans
101	Analysis based on past incidences
102	Early debriefing
103	Quickest possible return to normality
104	Quick return to normality—ongoing care for normal patients

Lessons learned	Communication
Pre-incident	
1	Terror awareness—train the public—communicate
2	Establish Improved alert system
3	Public engagement and empowerment—communication and teaching
4	Clear communication, organization and decision making skills
During the incident	
6	Delays in communication should be expected
7	Radio Equipment vital as often all other communication lines lost
8	Importance of reliable information
9	Effective intra-hospital communication
10	Constant update on resources and limitation of all hospitals
11	Better communication between disaster agencies
12	Importance of communication between different rescue teams
13	Identification vests help communication and command structures—clear roles
14	Intra and interhospital communication is important
15	Importance of public communication centre
16	Communication between disaster scene/EMS and hospital is often big problem
17	Use of protected phone lines and walkie-talkies
18	Early information/communication from site to assess severity
19	Early on radio/bleep system—later use of mobile phones possible
20	Clear, well-structured communication and coordination
21	Increase supplies through early communication with vendors
22	Bleeps and cable phones as cell service is often unreliable
23	Multiple scenes create difficult command and communication problems
24	Communication between rescue services is vitally important
25	Do not solely rely on mobile phones—danger of collapse
26	Establish a public information centre close to hospital
27	Use robust communication methods
28	Communication lines often fail—be prepared
29	Management of uninjured survivors and relatives—good communication
30	Concentrate initially on relaying as much information as possible
31	Important information: (1) the nature of the event (2) the estimated number and severity of casualties; (3) the exact location of the event; (4) the primary routes of approach and evacuation; (5) estimated time of arrival at the nearest hospital
32	Use megaphones if adequate
33	Turn off all non-critical mobile cell phones during terror event (government implementation)
34	Communication centre for relatives
35	No media inside hospital—media centre set up
36	Importance of communication mechanisms during terror
37	Communication with public—use of media
38	Good telecommunication system—with backup options
39	Create database of victims/casualties
40	Importance of communication/coordination between incident site and hospitals
41	Importance of even distribution between hospitals—communication
42	Early press briefings to stop hysteria
43	Communication failure will always happen
44	Good care despite communication failure—hence senior well-trained personnel
45	Communication-use of standardised operational terms
46	Good in-hospital communication between specialties
47	Decision making without all information—lack of communication unavoidable
48	Public Reassurance through good communication
49	Restricted internet access to avoid breakage
50	Communication with relatives
51	Better communication of patient information between prehospital and hospital setting
52	Communication channel between police, EMS and hospitals
53	Public relations and communication
54	Readiness of hospitals—good communication and preparation
55	Mutual communication systems
56	Better Integration of operators of different rescue chains + communication
57	Provide patient lists to police to ease communication/information gathering for relatives
58	Importance of patient hand over communication
59	Effective communication—improve information sharing
60	Sharing of corporate knowledge—communication of information
61	Good communication and situational awareness—use liaison officers
62	Media policy and communication—robust and well informed
63	Consider radio control mechanisms
64	Confidentiality when it comes to communication with media
65	Security and privacy issues when it comes to media communication
66	Quick and clear communication with relatives—to avoid information gathering via social media

Lessons learned	Preparedness/planning/training
Pre-incident	
1	Practise/drill—important!
2	Terror awareness—train the public
3	Trained prehospital personnel is a crucial factor
4	Update disaster plans—train them
5	Different sort of drills to prepare (manager drills/full scale drills)
6	Training is most important
7	Have and follow a pre-existing plan—based on experience
8	Thorough good quality preparation
9	Good prehospital care systems improve survival
10	Training of triage to reduce over and under triage
11	Debrief early and in a structured way
12	Preparation for incidents and injury types
13	Be prepared: have 1–3 months supply of surgical disposables
14	All hospitals should be included in contingency planning
15	All hospitals should be prepared to act as evacuation hospital—drills and training
16	Importance of damage control concepts—training
17	Cancellation of all elective surgical procedure
18	Emptying of ICU and wards
19	Importance of planning, coordination, training, financial support and well equipped medical services
20	Clear out hospital during latent phase
21	Have a major incident plan—have it rehearsed
22	Analysis based on past incidences
23	Analysis of gaps between scenario and response needed
24	Pre-event preparedness crucial—extensive planning improve outcome
25	Train core of nurses in emergency medicine skills
26	Have an emergency plan even if not a level one trauma centre
27	Rehearsal of emergency plan
28	Every hospital should be prepared for a major incident with terrorist background -solid emergency plans in situ
29	Importance of thorough analysis and short fallings
30	Good mix between planning and improvisation
31	A major incident plan is necessary—on a local as well as regional level
32	Meticulous planning
33	Extensive education
34	Regular review of the contingency plans
35	Emergency and disaster preparation and planning is crucial
36	All hospitals should be ATLS trained and have major incident drills
37	Regional major incident plan to help allocate resources
38	Have and activate contingency plans soon
39	Be prepared for uncertainty and unsafe environment
40	Having experience best preparation for next incident
41	Training saves lives
42	Drills based on past experiences
43	Teaching/training/education—best preparation
44	Disaster training best preparation for reality—systematic multidisciplinary training/drills
45	Train for new pattern of injuries
46	Readiness of hospitals—good communication and preparation
47	Public engagement and empowerment—communication and teaching
48	Staff training in combat medicine—cooperation with the military
49	Greater investment, integration, standardisation of disaster medicine
51	Multidisciplinary training—including police/fire service
52	Monthly multidisciplinary trauma training
53	Train the public/police in first aid/bleeding control
54	Importance of evaluation and improvement of emergency plans
55	Emergency preparedness based on planning/training/learning
56	Competence through continuous planning/training/drills
57	Cooperation: teaching of medical staff by military
58	Teaching of trauma management to med students
59	Therapy of paediatric cases—training is essential
60	Anticipation and planning—Plan Blanc obligatory
61	Awareness of tactical threat—idea of hazardous area response team
62	Training in trauma management
63	Planning and training—the value of organised learning
64	National process for debriefing and lessons learned
65	Regional standards for training

Lessons learned	Command
During the incident	
1	Strict command and control structures with designated hierarchy
2	Establish incident command system/centre—this is important
3	Early command and control structure—be prepared to rebuild
4	Avoid improvisation in command structure
5	Identification vests help communication and command structures—clear roles
6	Most senior medical officer = commander
7	Prompt and vigorous leadership
8	Civil defence coordinates and has overall command—clear structure
9	Importance of chain of command
10	Command structures—medical director vs administrative director
11	Incident commander appoints: liaison officer; public information officer; personnel officer; logistics officer; data officer; medical command officer; patient/family information officer
12	Chain of command: most senior official from all important specialties plus hospital admin
13	Multiple scenes create difficult command and communication problems
14	Have experienced decision maker
15	Command and control—regular trauma meetings
16	Importance of EMS command centre
17	Accept chaos phase—command structures will follow
18	Importance of local command structures—most senior official = commander in chief
19	Communication/cooperation between managers of different EMS
20	Work within established command and control structures
21	Clear distinction between command/control and casualty treatment
22	Lead by senior clinicians
23	Effective decision making—command is crucial
24	Command structures need to be robust
25	EMS command structures are vital
26	Dual command—ambulance/tactical commander vs medical commander
27	Command and control vs collaboration—both important
28	Flexible leadership
29	Leadership through ER physicians
30	Central Command—Health emergencies crisis management centre
31	Central command in hospital—director of medical operations
32	Good crisis management/command important
33	Multidisciplinary management
34	Clear communication, organization and decision making skills vital
35	Robust and simple organisation and command
36	Crisis management based on knowledge and data collection
37	Solid command structures and leadership based on experience and knowledge
38	Tactical management—get an overview and do not get stuck in details
39	Leadership/coordination through experienced healthcare professionals
40	Tactical command post in safe zone

Lessons learned	Triage
Pre-incident	
1	Establish national triage guidelines
2	Improve triage skills
3	Reproducible triage standards
4	Triage according to three ECHO—coloured cards
5	Casualty disposition framework with an effective enhanced triage process
During the incident	
6	Priority is quick triage, evacuation and transport to hospital
7	Establish casualty collection points/triage simple and early
8	Multiple triage areas—staff with freelancers
9	Coloured tags for triage
10	Use START system—simple triage rapid treatment
11	Doctors not deployed in red zone -triage in safe zone
12	Triage by most senior personnel
13	In-hospital triage according to ATLS
14	Systematic planning for triage, stabilisation and evacuation to hospital through chain of treatment stations
15	Triage at a distant site to disaster
16	Importance of triage—good triager—absolute authority
17	Deploy small medical teams for 2nd triage
18	Senior general surgeon triages at hospital entrance
19	Triage on arrival at hospital entrance as prehospital triage not necessarily reliable
20	Rapid primary triage—evacuation of the critical ill to nearest hospital (evacuation hospital) for stabilisation
21	Beware of undertriage
22	Importance of triage at incident site
23	Importance of retriage at hospital
24	Importance of triage concepts in general—avoid undertriage
25	Primary in-hospital survey through surgeons and anaesthetists
26	Diligence in triage
27	Large amount of over triage—no negative consequences/overtriage does not kill
28	Establishment of triage areas in hospital
29	Tertiary survey day after
30	Repeated effective triage maintains hospital surge capacity
31	Idea to establish triage hospital
32	Rapid primary survey and triage—delay of secondary survey
33	Most senior emergency physician directs traffic/surgeons overseas area—triage not by most senior personnel
34	Prehospital as well as hospital triage is vitally important
35	Importance of good primary triage
36	Frequent reassessment and triage
37	Quick triage—scoop and run—repeated triage at hospital
38	Quick effective good basic triage—reduction of overtriage
39	Improved triage through physician/paramedic teams
40	Enough equipment but mainly quick triage and transport
41	Deliberate overtriage
42	Directed quick patient flow to relieve triage area
43	Inadequate triage results in critically injured patients—retriage is vital
44	Outside triage area—not in hospital
45	Triage: absolute vs relative emergencies
46	Crisis teams to organise triage
47	Continuous retriage—similar triage system preclinical and in hospital
48	Triage outside hot zone—no therapy in hot zone if not trained
49	Most important triage point: able to walk vs not able to walk

Lessons learned	Staffing
Pre-incident	
1	Deploy trained prehospital personnel
2	Staff imprints lessons from mini-disasters and use this experience
3	Establishment of human resource pools—especially with volunteers
4	Too few nurses—improve incentives
5	Description of relevant capabilities of the medical system
6	Staff training in combat medicine—cooperation with the military
7	Up-to-date list of available staffing important
During the incident	
8	Descale as soon as possible → rest time for staff
9	Staff Safety is a major concern
10	Freelancers are important but difficult to manage
11	Multiple triage areas—possible staffing with freelancers
12	Quick response—increase staffing as soon as possible
13	Maximal increase of staffing needed—most important factor
14	Forward deployment of anaesthetist—allows for continuity of care
15	Relieve staff after 8–12 h for breaks
16	Optimise utilisation of manpower and supplies
17	Primary survey through surgeons and anaesthetists
18	ED staffed with nurse/doctor combo at each bed
19	Gather information and personnel during latent phase
20	Helicopters to transport staff and equipment
21	Triple: anaesthetist trauma surgeon abdominal surgery lead assessment and allocation to definite care
22	Efficient staff allocation
23	Pre hospital physicians useful
24	Using tags for triage—no resuscitation efforts until enough staffing
25	Train core of nurses in emergency medicine skills
26	Different specialties (ENT/psych) needed
27	Spread out teams to attend more patients
28	Too much staff available in ER—overcrowding
29	Good care despite communication failure—hence senior well trained personnel
30	Triage by senior medical officers
31	Keep track of staff showing up
32	Keep personnel in reserve/on standby
33	Experienced staff is vitally important
34	Surge in equipment and staff vital
35	Safety of personnel—idea of SWAT paramedics—therapy under fire
36	Increase blood bank staff
37	Photography staff/service to document injury
Post-incident	
38	Follow up on personnel—psychological and physiological

Lessons learned	Patient flow and distribution
Pre-incident	
1	Large number of mildly injured patients need to be expected and swiftly dealt with
2	Provide enough equipment but tailor to quick triage and transport
During the incident	
3	Majority of survivors are self-rescuer
4	Establish safe way for self-rescuer/non invalid patients
5	Increase ICU capacity move patients and unlock new areas
6	Patient flow—division between different hospital to avoid overload/right patient to the right hospital
7	Fast forward casualty flow
8	Coordinated distribution of casualties to hospitals
9	Log of most severely injured patients and their whereabouts
10	Quick redistribution of patients to clear ER for new ones
11	Use recovery room for monitoring unstable patient
12	Second wave of patient transfer between hospitals to avoid resource overstretching
13	Misdistribution between hospitals is a huge problem
14	Unidirectional patient flow—quick emptying of ED—one way pathway of care
15	Walking wounded redirected to satellite areas
16	Early evaluation of patients by senior doctors—early estimation of ICU capacity/operating capacity needed
17	Transport off ICU patients to different hospitals needs to be thought of
18	Rapid removal from critically ill patients out of an unsafe environment
19	Transferring patients rapidly to definite care—rapid scene clearance
20	Consider the need for secondary transport (interhospital)
21	Distinction between circle 1 and circle 2 hospitals—direction of casualties accordingly
22	Quick evacuation of casualties—if stable enough severely injured patients to trauma hospitals
23	ED as epicentre—clear ED quick
24	Establish different treatment areas: fast track, psychiatric, major trauma, etc.
25	Primary evacuation of mildly injured patients to distant hospitals
26	Treat patient in level 2 trauma centres and only transfer if necessary to level 1 trauma centres
27	Divert non urgent patients to hospitals further away from incident site
28	Survivor reception centres to alleviate hospitals
29	Primary and balanced distribution between hospitals
30	Timely evacuation out of unsafe zone
31	Overload of patients at close by hospitals is huge problem
32	Fast track route for minor injuries
33	Patient flow—evacuation to cold zones
34	Directed quick patient flow to relieve triage area
35	Secondary patient flow according to capacity and specialty
36	Relocation of current patients
37	Cooperation between hospitals and trauma centres—recognise your limits and transfer
38	Tourniquet use und quick transfer to definite care
39	Track patients through hospital is a difficult task
40	Casualty clearing station—part of patient flow
41	Casualty disposition framework with an effective enhanced triage process
42	Safe transfer and handover of existing patients

Lessons learned	Cooperation and multidisciplinary approach
Pre-incident	
1	Common goal is an important benefit
2	Cross organisational planning important
3	Communication channel between police, EMS and hospitals
4	Staff training in combat medicine—cooperation with the military
5	Awareness of tactical threat—idea of hazardous area response team
6	Sharing of corporate knowledge—communication of information
7	Clinical representation at strategic level to facilitate cooperation between networks/regions
8	Simultaneous search/rescue/treatment
During the incident	
9	Better communication between disaster agencies is important
10	Importance of communication between different rescue teams
11	Especially trauma patients need teamwork and good cooperation (surgery/anaesthetic)
12	Cooperation of the entire medical system—prehospital as well as hospital
13	Increase supplies through early communication with vendors
14	Collaboration with police to deliver supplies
15	Police command centre within hospital
16	Chain of command: most senior official from all important specialties plus hospital admin
17	Communication between rescue services vitally important
18	Good teamwork is crucial
19	Triple: anaesthetist, trauma surgeon abdominal surgeon lead assessment and allocation to definite care
20	Multidisciplinary meetings
21	Most senior emergency physician directs traffic/surgeons overseas area—triage not by most senior personnel
22	Flexibility of services important—interaction/cooperation important
23	Possibility for emergency services to cooperate and communicate
24	Combined activation of major incident plans (all EMS services)
25	Joint field command post
26	Cooperation and communication between hospitals and all emergency services
27	Dual surgical command-triage
28	Cooperation between police and EMS
29	Methodical multidisciplinary care delivery
31	Good cooperation/collaboration between services is vital
32	Good interdisciplinary cooperation is vital
30	Command and control vs collaboration—both important
33	Multidisciplinary care saves lives
34	Cooperation between EMS and police/fire services
35	Multidisciplinary training—including police/fire service
36	Multi-professional networks/interaction including mental health
37	Cooperation between hospitals and trauma centres—recognise your limits and transfer
38	Crucial interaction/communication between hospital/police/municipalities
39	Provide patient lists to police to ease communication/information gathering for relatives
40	Good communication between incident site and hospital
41	Law enforcement medical commander—cross over between specialties/cooperation
42	Cooperation between civilian rescue teams and military
43	Good communication and situational awareness—use liaison officers
44	Coordination and collaboration should be planned and practised at intra/inter-regional, multiagency and multiprofessional levels
45	Support from neighbouring regions during terror

Lessons learned	Equipment and supplies
Pre-incident	
1	Functioning equipment is vitally important (broadband internet)
2	Constant resource evaluation
3	Combat medical care—reduced level of treatment per patient due to resource insufficiencies
4	Need for appropriate equipment + supplies
5	Increase supply of available blood products
6	Mobile multiple casualty carts and disaster supply carts with equipment are helpful
7	Increase supplies through early communication with vendors
8	Assess Need for chemical and radiological monitors
9	Description of relevant capabilities of medical system
10	Provide megaphones
11	Provide protective personal equipment
12	Install mobile mass casualty vehicles with additional supplies
13	Increase and storage of supplies
14	Supply chains need to be reliable/organised well
15	Regional major incident plan to help allocate resources
During the incident	
16	Restrict laboratory and radiology testing
17	Protection of medical assets
18	Increase equipment—prep minor OR for major casualties
19	Rapid primary triage—only evacuation of the critical ill to nearest hospital (evacuation hospital) for stabilisation—to avoid resource overstretching
20	Second wave of patient transfer to avoid resource overstretching
21	Optimise utilisation of manpower and supplies
22	Collaboration with police to deliver supplies
23	Helicopters to transport staff and equipment
24	Basic equipment important and needed
25	Use of radio systems
26	Basic first aid kits on buses/trains
27	Allocation of resources difficult especially with multiple incidents
28	Enough equipment but mainly quick triage and transport
29	More advanced equipment including CBRN
30	Allocate resources to correct diagnosis
31	Extensive use of tourniquet
32	Challenge of technology-equipment may fail
33	Back up resources—mobilise equipment and staff
34	Use of clotting devices/tourniquet
35	Surge capacity in equipment and staff is vital
36	Avoid main gate syndrome—overwhelmed resources at the closest hospital
37	Regional resource mobilisation is vital

Lessons learned	Medical treatment + type of injury
Pre-incident	
1	Use critical mortality rate as indicator for assessing medical care
2	Terror attack cause different/specific injury patterns
3	Except many blast injuries
4	Average ISS Score of ICU admission
5	Professional abilities are important
6	Train for new pattern of injuries
7	Medical management and knowledge vitally important
8	Stop the bleeding—tourniquet use—train as basic first aid
9	Integration of TCCC to ATLS
10	Improve therapy of paediatric cases—training
During the incident	
11	Evacuate patients as soon as possible
12	Rapid treatment is important
13	Use START system—simple triage rapid treatment
14	Combat medical care—reduced level of treatment per patient due to resource insufficiencies
15	Early aggressive resuscitation predicts survival
16	Available surgical capacity needs to be increased
17	Restrict laboratory and radiology testing—minimal investigations
18	Only damage control surgery—the rest must wait
19	Medical treatment dependent on type of attack
20	Rapid provision of definite care
21	Therapy according to ATLS guidelines
22	Predominance of minor injuries during terrorist bombings (secondary/tertiary blast effect) and worried well patients
23	Critical injury appears roughly in 1/3rd of the cases
24	Blast injury results often in immediate death—if not there is often a combination with ear injury
25	Only 5% ISS > 15; 2% ISS > 25
26	Main injuries: blunt trauma, blast injury, penetrating wounds, burns
27	Rapid removal from critically ill patients out of an unsafe environment—scoop and run Therapy
28	Damage control treatment and mind set to increase surge capacity
29	Using tags for on scene triage—no resuscitation efforts until enough staffing
30	Treat patient in level 2 trauma centres and only transfer if necessary to level 1 trauma centres
31	Damage control treatment—no provision of individual definite care
32	Use ATLS/PHLTS standards
33	Use tactical combat casualty care + haemorrhage control
34	Roughly 10% suffer major injury
35	Schedule operations according to urgency
36	Extensive use of tourniquet
37	Offer immediate access to OR
38	Patient therapy/flow: tourniquet use und quick transfer to definite care
39	Safety vitally important—extent of therapy based on situational safety

Lessons learned	Zoning and scene safety
Pre-incident	
1	Full personal protective equipment and knowledge of the prehospital environment helpful
2	Beware of hospitals being soft targets
3	Safety of personnel—idea of SWAT paramedics—therapy under fire
4	Awareness of tactical threat—idea of hazardous area response team
During the incident	
5	Security at all hospital entrances—consider immediate lockdown
6	Simultaneous search/rescue/treatment—beware of security risks of this concept
7	Scene safety and scene control—beware of loss of rescue personnel—safety first
8	Beware second hit principle—protect trained personnel
9	Establish a safe way for self-rescuer
11	Safety of staff paramount
12	Rapid removal from critically ill patients out of an unsafe environment
13	Scene safety—important but huge problem hence rapid evacuation
14	Awareness for explosive devices being carried into hospital
10	Doctors not in red zone—triage in safe zone
15	Continuous assessment of scene safety
16	Safety first—triage/command outside danger zone
17	Manage uncertainties and scene
18	Evacuation problematics due to scene and geographical environment
19	Importance of scene safety and terror control
20	Scene safety—secondary attack/collapsing buildings/explosive Device
21	Conventional rescue teams out of danger zone
22	Operating capacity within on scene dressing station-tactical physicians as concept
23	Scene safety—zoning (exclusion zone)
24	Scene safety: develop best compromise btw safety of responders, immediate care and fast extraction
25	Triage outside hot zone—no therapy in hot zone if not trained
26	Tactical command post in safe zone
27	Scene **s**afety cannot be guaranteed
28	Safety vitally important—extent of therapy based on situational safety
29	Challenges of being in the hot zone—multifaceted and continuously evolving
30	Recognition of situational aspect and severity + complexity—evolving risk

Lessons learned	Psychiatric support
Post-incident	
1	Early psychiatric help is important
2	Site for acute stress disorder therapy needed
3	Good psychological support is necessary and important
4	Importance of post-traumatic stress disorder treatment groups
6	Do not underestimate the psychological and physical effects on health care workers
7	Psychological support for emergency services/healthcare worker/staff
8	Debriefing as stress relief
9	Psychiatric support before discharge for all patients
10	Psychological support for mildly injured patients
11	Set up survivor groups/psychological support
13	Psychological support to reduce long term impact of terrorism
14	Establishment of mental health counselling for staff
15	Psychiatric illness as hazard for emergency personnel
16	Establish psychological support centre
17	Low PTSD with good preparation, debriefing and high role clarity
18	Psychological follow up for staff and patients
19	Multiprofessional networks/interaction inclusive Mental Health
20	Everyone should be seen by psychiatric experts
21	Psychological care—Increase psychological support short and long term
22	1/3 of victims develop post traumatic stress disorder (PTSB)
23	Psychological support—informal and formal Treatment
24	Improve bereavement support
25	Psychological first aid approach including self help
26	Bereavement nurses—24/7 access in the first 48 h
27	Monitor high risk groups of PTSD

Lessons learned	Record keeping
Pre-incident	
1	Create database of victims/casualties
2	Identification difficulties of victims—improve documentation to allow quicker identification
3	Improvement in identification: INTERPOL Disaster Victim Identification Standard
4	Standardised documentation at regional level/need for national casualty identification system
5	Patient identification difficult task—standardized identification and documentation systems
During the incident	
6	Written documentation strapped to patient
7	Early start of data collection
8	Good record keeping is essential
9	Lead agency to solely deal with record keeping
10	Importance of data collection of casualties at the scene
11	Importance of documentation—which patient has already been triaged
12	Better communication of patient information between prehospital and hospital setting
13	Detailed documentation of the disaster operation
14	Crisis management based on knowledge and data collection
15	Track patients through hospital—this is a difficult task
16	Photography staff/service to document injury
17	Importance of patient identification to allow for family reunification/bereavement

Lessons learned	Role understanding
1	Clear identification methods of roles—tags/vests—helps communication and command structures
2	Dedicated roles with clear defined duties during event—command and control physician; discharge/ patient flow organiser; ED supervisor
3	Assigned roles in disaster plan
4	Flexibility but clear roles
5	Know your capabilities/professional role
6	Low post traumatic stress disorder with good preparation, debriefing and high role clarity
7	Clear defined roles help to give security and confidence and improve outcome

Lessons learned	Team spirit
1	Keep team spirit up
2	Form coalition to keep up spirit and improve
3	Staff solidarity and professionalism vital
4	Public engagement and empowerment—communication and teaching
5	Professionalism and team spirit important for success
6	Mutual support important

## Discussion

This systematic review is the first of its kind to review the vast amount of literature dealing with lessons learned from terror attacks. It thus contributes to a better understanding of the consequences of terror attacks since 2001. It also brings order to the multitude of defined lessons learned and allows for an overview of all the important findings.

Our data has shown that, despite the difference in attacks, countries, social and political systems and casualties involved, many of the lessons learned and issues identified are similar. Important to note was the fact that time of article release did not relate to content. Many articles written after the London attacks in 2005 formulated similar if not the same lessons learned as articles written in 2017 about Utoya [36, 52]. This is a major point of concern as it indicates, that despite the knowledge about the issues and the existence of already developed, excellent concepts [56, 79, 80], their successful implementation and continuous improvements seem to be lacking.

One of these well-developed concepts, the Tactical Combat Casualty Care (TCCC), began as a special operations medical research programme in 1996 and is now an integral part of the US Army's trauma care [79]. The Committee on TCCC, which was established in 2001, ensures that the TCCC guidelines are regularly updated [79]. Many of the lessons learned listed in our review are an integral part of these guidelines and are addressed with concrete options for action. For Example, the principles of Tactical Evacuation Care provide detailed instructions on the management of casualties under the special conditions of evacuation from a danger zone [81]. Moreover, the lack of knowledge on how to deal with injuries caused by firearms or explosive devices, which was mentioned in many articles, could be remedied by a consistent integration of the TCCC guidelines into the training and drills of emergency service staff.

Another concept that deals with the management of terrorist attacks and mass shootings is the Medical Disaster Preparedness Concept “THREAT”, which was published after the Hartford Consensus Conference in 2013 [56]. The authors defined seven deficits as lessons learned and recommended concrete measures to address them. These lessons were included in our review and were mentioned in one form or the other in many of the articles. The defined THREAT concept components were:

- T: Threat suppression
- H: Haemorrhage control
- RE: Rapid extraction to safety
- A: Assessment by medical providers
- T: Transport to definitive care.

Consistent implementation of these points in training and practice would be an important step towards improving preparation for terror attacks.

A good example of the successful implementation of an interprofessional concept is the 3 Echo concept (Enter, Evaluate, Evacuate) [80]. It was developed and introduced with the goal to optimize the management of mass shooting incidents. At the beginning of concept development stood the identification of deficits. Those deficits correspond to those that we found in the presented systematic review. The introduction of the concept in training and practice has led to successful management of a mass shooting event in Minneapolis, Minnesota, USA in 2012 [80]. This outlines once again the importance of translating lessons learned into concrete concepts, to integrate them into the training and to practice them regularly in interprofessional drills. Just as the 3 Echo concept is based on interprofessional cooperation, the Joint Emergency Services Interoperability Principles (JESIP) project is also based on this principle [82]. It is the standard in Great Britain for the interprofessional cooperation of emergency services in major emergencies or disasters. Through simple instructions and a clear concept, both the aspect of planning and preparation as well as the concrete management of operations are taken care of [82].

In interpreting the lessons learned in this systematic review, the question arises whether they are specific to terrorist attacks. Our review deals exclusively with lessons learned from terrorist attacks. Other publications, however, systematically addressed the management of terrorist and non-terrorist mass shootings and disasters. Turner et al. reported the results of a systematic review of the literature on prehospital management of mass casualty civilian shootings [83]. The authors identified the need for integration of tactical emergency medical services, improved cross-service education on effective haemorrhage control, the need for early and effective triage by senior clinicians and the need for regular mass casualty incident simulations [83] as key topics. Those correspond congruently with the lessons learned from terrorist attacks that were found and presented in this systematic review.

Hugelius et al. performed a review study and identified five challenges when managing mass casualty incidents or disaster situations [84]. These were “to identify the situation and deal with uncertainty”, “to balance the mismatch between contingency plan and reality”, “to establish functional crisis organisation”, “to adapt the medical response to actual and overall situation” and “to ensure a resilient response” [84]. The authors included 20 articles, of which 5 articles dealt with terror and mass shooting (including the terror attacks in Paris and Utoya). Although only 25% of the included articles dealt with terrorist attacks, the lessons learned are again very comparable to the results of this systematic review.

Challen et al. published the results from a scoping review in 2012 [11]. The authors stated that “although a large body of literature exists, its validity and generalisability is unclear” [11]. They concluded that the type and structure of evidence that would be of most value for emergency planners and policymakers has yet to be identified. If trying to summarise the development since that statement it can be assumed that on one hand sound concepts have been developed and implemented. On the other hand however, the lessons learned in recent terror attacks still emphasize similar issues as compared to those from the beginning of the analysis in 2001, showing that there is still work to be done. It should be mentioned at this point, that there was a federal conducted evaluation process in Germany after the European terror attacks in 2015/2016. The lessons learned were published in 2020 by Wurmb et al. and were very comparable to those of this systematic review [85]. Furthermore the terror and disaster surgical care (TDSC^®^) course was developed in 2017 by the Deployment, Disaster, Tactical Surgery Working Group of the German Trauma Society to enhance the preparation of hospitals to manage mass casualty incidents related to terror attacks [86]. Finally it is important to mention, that hospitals and rescue systems must prepare not only for terrorist attacks, but also for a wide spectrum of disasters. Ultimately, this is the key to increased resilience and successful mission management.

## Limitations

This systematic review has several limitations. Due to the vast amount of information only PubMed was used as a source. From the authors' point of view, this is a formal disadvantage, but it does not change the significance of the study as in contrast to the question of therapy effectiveness or the comparison of two forms of therapy, the aim here is to systematically present lessons learned. To get even more information, the data search could have been extended to other databases (e.g. Cochrane Library, Web of science) and the grey literature. Given the number of included articles, it is questionable whether this would have significantly changed the central message of the study. It is even possible that this would have made a systematic presentation and discussion even more difficult. CBRN attacks have been excluded from the research. The reason for that was that many special aspects have to be taken into account in these attacks. Nevertheless CBRN attacks are an important topic, which would need further exploration in the future. The restriction to OECD countries certainly causes a special view on the lessons learned and is thus also a source of bias. However, the aim was to look specifically at countries where terror attacks are a rather rare event and rescue forces and hospitals are often unfamiliar with managing these challenges. Special injury patterns associated with terror attacks were not considered. This reduces the overall spectrum of included articles, but from the authors' point of view, a consideration of these would have exceeded the scope of this review.

## Conclusion

The first thing that stands out is that most lessons learned followed a certain pattern which repeated itself over the entire time frame considered in the systematic review. It can be assumed that in many cases it is therefore less a matter of lessons learned than of lessons identified. Although sound concepts exist, they do not seem to be sufficiently implemented in training and practice. This clearly shows that the improvement process has not yet been completed and a great deal of work still needs to be done. The important practical consequence is to implement the lessons identified in training and preparation. This is mandatory to save as many victims of terrorist attacks as possible, to protect rescue forces from harm and to prepare hospitals and public health at the best possible level.
